# Sustained Inflation Reduces Pulmonary Blood Flow during Resuscitation with an Intact Cord

**DOI:** 10.3390/children8050353

**Published:** 2021-04-29

**Authors:** Jayasree Nair, Lauren Davidson, Sylvia Gugino, Carmon Koenigsknecht, Justin Helman, Lori Nielsen, Deepika Sankaran, Vikash Agrawal, Praveen Chandrasekharan, Munmun Rawat, Sara K. Berkelhamer, Satyan Lakshminrusimha

**Affiliations:** 1 Department of Pediatrics, University at Buffalo, Buffalo, NY 14203, USA; lauren.davidson26@gmail.com (L.D.); sfgugino@buffalo.edu (S.G.); carmonko@buffalo.edu (C.K.); jhelman@buffalo.edu (J.H.); lnielsen@buffalo.edu (L.N.); dragrawalmd@gmail.com (V.A.); pkchandr@buffalo.edu (P.C.); munmunra@buffalo.edu (M.R.); berkelsa@uw.edu (S.K.B.); 2 Buffalo Neonatology Associates, Sisters of Charity Hospital, Buffalo, NY 14214, USA; 3 Department of Pediatrics, University of California at Davis, Davis, CA 95616, USA; dsankaran@ucdavis.edu (D.S.); slakshmi@ucdavis.edu (S.L.); 4 Department of Pediatrics, Loma Linda University, Loma Linda, CA 92350, USA; 5 Department of Pediatrics, University of Washington, Seattle, WA 98195, USA

**Keywords:** sustained inflation, delayed cord clamping, placental transfusion, pulmonary blood flow, perinatal asphyxia

## Abstract

The optimal timing of cord clamping in asphyxia is not known. Our aims were to determine the effect of ventilation (sustained inflation–SI vs. positive pressure ventilation–V) with early (ECC) or delayed cord clamping (DCC) in asphyxiated near-term lambs. We hypothesized that SI with DCC improves gas exchange and hemodynamics in near-term lambs with asphyxial bradycardia. A total of 28 lambs were asphyxiated to a mean blood pressure of 22 mmHg. Lambs were randomized based on the timing of cord clamping (ECC—immediate, DCC—60 s) and mode of initial ventilation into five groups: ECC + V, ECC + SI, DCC, DCC + V and DCC + SI. The magnitude of placental transfusion was assessed using biotinylated RBC. Though an asphyxial bradycardia model, 2–3 lambs in each group were arrested. There was no difference in primary outcomes, the time to reach baseline carotid blood flow (CBF), HR ≥ 100 bpm or MBP ≥ 40 mmHg. SI reduced pulmonary (PBF) and umbilical venous (UV) blood flow without affecting CBF or umbilical arterial blood flow. A significant reduction in PBF with SI persisted for a few minutes after birth. In our model of perinatal asphyxia, an initial SI breath increased airway pressure, and reduced PBF and UV return with an intact cord. Further clinical studies evaluating the timing of cord clamping and ventilation strategy in asphyxiated infants are warranted.

## 1. Introduction

Birth asphyxia affects 4 million newborn infants worldwide each year [1,2]. Appropriate resuscitative measures result in a substantial reduction in birth asphyxia-associated mortality and morbidity [3]. Establishing early and effective ventilation is key to successful neonatal resuscitation. Every 30 s delay in the initiation of ventilation increases the risk for early death and morbidity [4]. Sustained inflation (SI) may facilitate the early establishment of functional residual capacity (FRC). Revised European resuscitation guidelines recommend maintaining inflation pressure for 2–3 s for the first five inflations in apneic infants [5]. Minimal evidence exists for its use in term infants. A single SI immediately after birth improved the speed of circulatory recovery and lung compliance in the resuscitation of near-term asphyxiated lambs [6]. SI with continuous chest compressions is being evaluated as a resuscitation strategy in animal studies [7,8].

Available literature on SI is in neonatal/animal models with immediate or early cord clamping (ECC). ECC in asphyxia compromises both sources of LV preload (pulmonary venous (PV) return and umbilical venous (UV) return through the foramen ovale), resulting in decreased perfusion [9]. However, because of a lack of data demonstrating benefits and limitations of performing extensive resuscitation with an intact cord, delayed cord clamping (DCC) is currently not recommended, but is being evaluated in randomized controlled trials (e.g., SAVE NCT04070560). There is little information on resuscitation with SI and an intact cord. Our objectives were to determine the effect of ventilation (SI vs. positive pressure ventilation—PPV) with early or delayed cord clamping on resuscitation parameters in asphyxiated, bradycardic, near-term lambs. We hypothesized that SI with DCC would result in improved pulmonary and cerebral hemodynamics and enhanced gas exchange.

## 2. Materials and Methods

Surgical Protocol: The study was approved by the Institutional Animal Care and use committee (IACUC) at the University at Buffalo and the methods were consistent with the NIH guide for the care and use of laboratory animals (NIH Publications No. 8023, revised 1978) and in accordance with the ARRIVE guidelines. Time-dated ewes at 142–145d gestation (Term 147d) from May Family Enterprises (Buffalo Mills, PA, USA) were fasted overnight, then induced for anesthesia with intravenous diazepam and ketamine. Ewes were intubated and ventilated with 2–3% isoflurane. In total, 28 fetal lambs were partially exteriorized, intubated and drained of excess lung fluid to simulate the decrease in lung liquid with labor. ETT was occluded to prevent air exchange with gasping. Fetal lambs were instrumented [10] with catheters placed in the right carotid artery (CA) and jugular vein (JV) for blood pressure measurements and blood sampling. Flow probes (Transonic Systems Inc., Ithaca, NY, USA) were placed around the left CA, left pulmonary artery and one umbilical artery and vein. Preductal pulse oximetry was measured using a Nonin pulse oximeter sensor (EQUANOX™, Nonin Medical Inc, Plymouth, MN, USA). Asphyxia was induced by umbilical cord occlusion using a vascular occluder until the mean blood pressure (MBP) was less than or equal to 22 mmHg, when the cord was released, and lamb was exteriorized. In a prior study, the target MBP < 20 mmHg resulted in a need for extensive resuscitation and high mortality [6]. By initiating resuscitation at a MBP of 22 mmHg, we anticipated an adequate degree of asphyxia without extensive mortality or the need for advanced resuscitative efforts.

**Figure 1:**
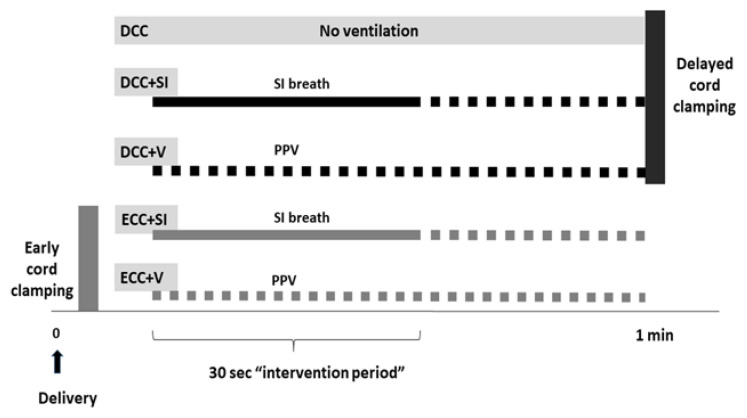
Study groups based on the timing of cord clamping and type of ventilation. DCC: Delayed cord clamping, ECC: Early cord clamping, SI: Sustained inflation, PPV: Positive pressure ventilation.

Randomization: Lambs were randomized prior to delivery to ECC (cord clamped immediately) and DCC (cord was clamped after 60 s). They were further randomized based on the timing of ventilation onset as well as the type of initial ventilation into five groups: ECC + V, ECC + SI, DCC, DCC + V and DCC + SI (Figure 1).

Resuscitation: A T-piece resuscitator was used for ventilation, with 21% O_2_ at a rate of 40 breaths/min, an initial peak inflation pressure (PIP) of 35 cm H_2_O and a peak end-expiratory pressure (PEEP) of 5 cm H_2_O during the intervention period for all groups. The two SI groups—ECC + SI and DCC + SI—were resuscitated with an initial SI breath with a T-piece resuscitator with a PIP of 35 cm H_2_O for 30 s. In the DCC group, ventilation was delayed until after the cord was clamped at 60 s. The intervention period was defined as the 30 s period when SI breath was delivered. In non-SI groups, the corresponding 30 s time period after the onset of resuscitation was taken as the intervention period. PIP was subsequently adjusted as needed to obtain an adequate chest rise, and PPV continued with 21% O_2_ according to neonatal resuscitation guidelines [11] at a rate of 40–60 breaths/min in all groups. If lambs went into asystole, resuscitation was continued with CC and 100% oxygen [11]. Epinephrine (0.01–0.03 mg/kg/dose) was administered intravenously at 3 min for asystolic lambs. Inspired oxygen was titrated to maintain the recommended preductal SpO_2_ [11]. Arterial blood gases were drawn at the initiation of resuscitation, then every minute for the first 5 min followed by every 5 min until the end of the study, and analyzed using a radiometer blood gas analyzer (ABL 800 FLEX, Copenhagen, Denmark). Prespecified hemodynamic parameters were recorded for a period up to 30 min from birth, following which the lambs were euthanized using pentobarbital per approved lab protocols. Physiological parameters including heart rate (HR), systemic BP and vascular flows were continuously recorded using AcqKnowledge Acquisition and Analysis Software (BIOPAC systems, Goleta, CA, USA). An end-tidal carbon dioxide adapter was attached to the ETT and a Philips NM3 monitor (Respironics, Murrysville, PA, USA) was used to measure airway pressure.

The magnitude of placental transfusion was assessed using biotinylated RBC to measure the red cell volume [12]. Next, 20 mL of maternal packed RBC was drawn a few days prior to the study and washed with a buffer solution three times. This was then divided into two groups; a low-density (LD) group of 12 ug/mL biotin and high-density (HD) group of 96 ug/mL biotin, both prepared with a target Hct of 50%. A baseline complete blood count (CBC) was drawn from the fetal lamb before injection. The low-density sample was infused into the lamb while still under maternal-fetal circulation, and samples were collected before and 10 min after injection to permit equilibration. This sample was used to measure the fetoplacental RBC volume. After delivery, the high-density biotin-labeled RBCs were injected to measure the newborn RBC volume. FICT-avidin (fluorescent marker) was added to the saved in vivo samples and measured with a flow cytometer. This labeled and counted the number of LD- and HD-labeled RBCs circulating in each sample to help determine the fetal vs. newborn total blood volume for each group studied.

The magnitude of placental transfusion was assessed between groups using the following equations.

(i) RBC volume = Amount of biotinylated RBC injected ÷ concentration of biotinylated RBC in the sample after equilibration
(ii) Blood volume = RBC volume × 100 ÷ Hct
(iii) Residual placental volume = Fetoplacental blood volume—neonatal blood volume
(iv) Fraction of neonatal retained blood = Neonatal blood volume ÷ Fetoplacental blood volume.

Primary end-points: The primary end-point was the time to circulatory stabilization, defined as the time taken to reach the baseline carotid blood flow—CBF (prior to umbilical cord occlusion), stable HR (≥100/min) and a MBP of ≥40 mmHg.

Secondary end-points: The secondary end-points included changes in systemic hemodynamics (CBF and MBP), left pulmonary blood flow (PBF) and umbilical arterial (UA) and venous (UV) flow changes during the 30 s intervention period between groups.

Statistical analysis and power calculation: Power and sample size calculations were based on a previous study of SI in asphyxiated near-term lambs [6], where the time to achieve circulatory stabilization within subject group was normally distributed with a standard deviation of 12 s. If the true difference in SI and PPV means was 25 s, we needed to study five subjects in each group to be able to reject the null hypothesis with a probability (power) of 0.8 and a Type I error probability of 0.05. Continuous variables were expressed as the mean and standard deviation (SD) and analyzed by ANOVA between groups with Fisher’s post hoc test. Categorical variables were analyzed using the chi-square test with Fisher’s exact test if appropriate. GraphPad Prism (San Diego, CA, USA) was used for statistical analysis. Statistical significance was defined as *p* < 0.05.

## 3. Results

Lambs were comparable in birth weight with similar baseline hemodynamic measurements that were obtained after instrumentation. The baseline UV flow was higher than the UA flow in most lambs. The extent of asphyxia was similar, as reflected by comparable pH and lactate levels (Table 1). Although intended as a model of bradycardia with perinatal asphyxia without cardiac arrest, 2–3 lambs in each group progressed to asystole similar to previously described studies in this model with cord occlusion [13].

### 3.1. Airway Pressure

As expected, the mean airway pressures in the SI groups, ECC + SI and DCC + SI, were significantly higher than in the PPV groups, ECC + V and DCC + V (Table 2), during the intervention period.

### 3.2. Primary Outcomes

No significant differences were noted among the five groups in time to reach baseline CBF, HR ≥ 100 bpm or MBP ≥ 40 mm Hg (Table 2).

### 3.3. Hemodynamics during Intervention Period

Among the DCC groups, the DCC + SI group had significantly decreased PBF compared to the DCC and DCC + V groups. MBP was comparable between groups (Table 2). Umbilical flows during the intervention period in all DCC groups were significantly lower compared to the baseline values prior to the onset of asphyxiation by cord compression. Significantly lower UV flow was seen during SI compared to DCC and DCC + V without any corresponding statistically significant differences in UA flow (Table 2).

### 3.4. Post Resuscitation Hemodynamics

**Figure 2:**
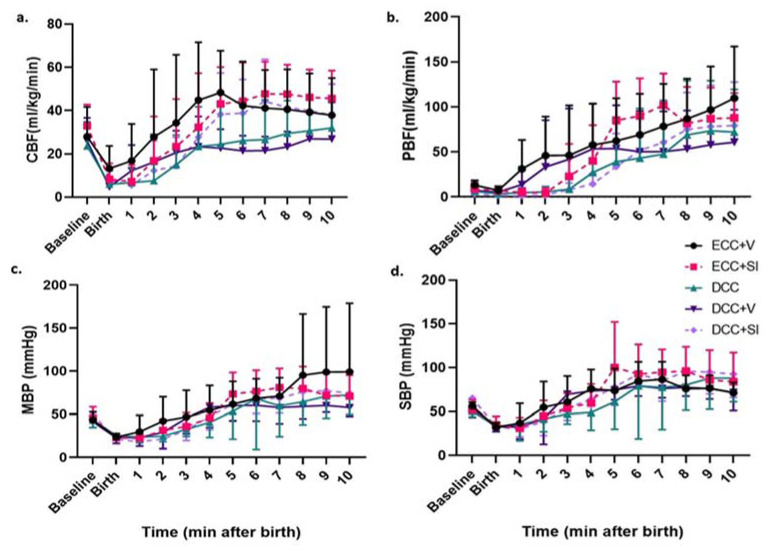
Post resuscitation hemodynamics in the first 10 min after birth in the five study groups: (**a**) carotid blood flow, (**b**) pulmonary blood flow, (**c**) mean blood pressure and (**d**) systolic blood pressure in all five study groups, with dashed lines reflecting the SI groups. Data are represented as mean ± SD. Black circle: ECC + V (*n* = 5), pink square dashed: ECC + SI (*n* = 6), green triangle: DCC (*n* = 5), purple triangle: DCC + V (*n* = 5), purple diamond dashed: DCC + SI (*n* = 7).

CBF decreased during asphyxia and increased after birth in all groups (Figure 2a), reaching baseline values by 5 min. No statistically significant differences in CBF patterns were noted among the five individual groups. However, ECC + V and ECC + SI appeared to result in a rebound of cerebral blood flow above baseline, while DCC (with and without ventilation) resulted in a steady increase back to baseline values without overshoot. PBF (Figure 2b) in all lambs increased after birth; however, at 1–3 min, the PPV groups (ECC + V and DCC + V) demonstrated a trend towards higher PBF, compared to the SI groups (ECC + SI and DCC + SI). MBP and SBP (Figure 2c,d) were similar in the 30 min period after birth, with figures depicting the first 10 min.

### 3.5. Gas Exchange and Lactate

Arterial pH, pCO_2_, pO_2_ and lactate were similar between groups at 2, 5 and 10 min, as were PaO_2_/FiO_2_ (P/F) ratios at 2 and 5 min. However, at 10 min, P/F ratios were significantly higher in the DCC + SI group along with a lower PaCO_2_. Among the DCC groups, the required FiO_2_ trended lower, and the P/F ratio was significantly higher in the DCC + SI group (Table 3).

### 3.6. Magnitude of Placental Transfusion

Residual placental volume and newborn blood volume were similar between the ECC and DCC groups (Table 4). Speculating that placental transfusion was interrupted during arrest, we performed a subgroup analysis of non-arrested lambs. The DCC lambs had a significantly higher fraction of fetoplacental volume and a decreased residual placental volume compared to the ECC lambs (Table 4).

### 3.7. Mode of Ventilation

**Figure 3:**
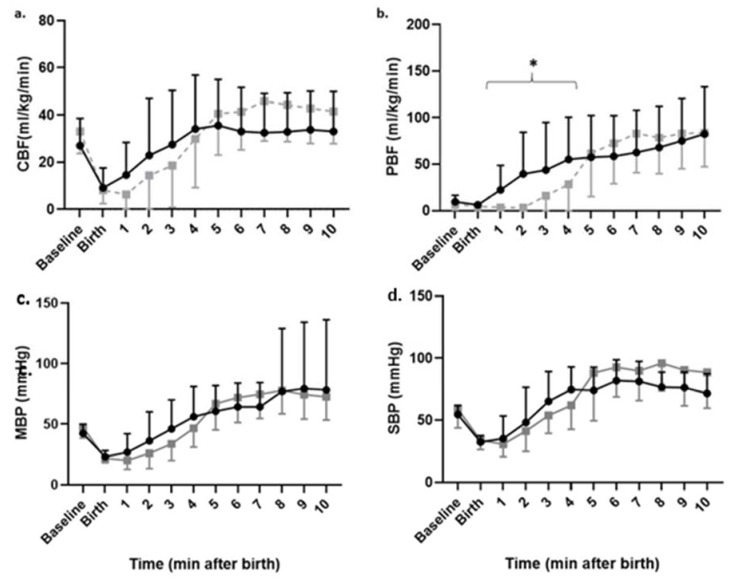
Post resuscitation hemodynamics in the first 10 min after birth in ventilation groups—effect of sustained inflation (gray squares, N = 13) is compared to positive pressure ventilation (Black circles, N = 10): (**a**) carotid blood (CBF), (**b**) pulmonary blood flow (PBF), (**c**) mean blood pressure (MBP) and (**d**) systolic blood pressure (SBP). Data are represented as mean ± SD. * *p* < 0.01 by repeated measures ANOVA.

Comparing SI (ECC + SI and DCC + SI) vs. PPV (ECC + V and DCC + V), the CBF patterns were not different (Figure 3a); however, the SI significantly reduced PBF in the first 4 min after birth (Figure 3b). MBP and SBP were similar with PPV or SI (Figure 3c,d).

### 3.8. Timing of Cord Clamping

**Figure 4:**
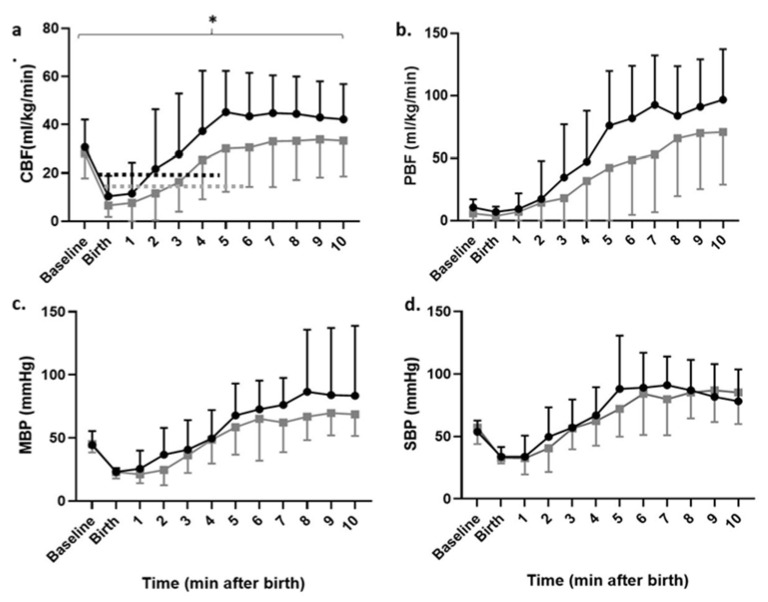
Post resuscitation hemodynamics in the first 10 min after birth based on the timing of cord clamping: (**a**) carotid blood flow, (**b**) pulmonary blood, (**c**) mean blood pressure and (**d**) systolic blood pressure. Data are represented as mean ± SD. * *p* < 0.05 repeated measures ANOVA. Black circles: ECC. Grey squares: DCC.

DCC reduced the CBF overshoot that was noted with ECC during the post resuscitation period (Figure 4a). Both groups had similar PBF, MBP and SBP post resuscitation (Figure 4b–d).

## 4. Discussion

**Figure 5:**
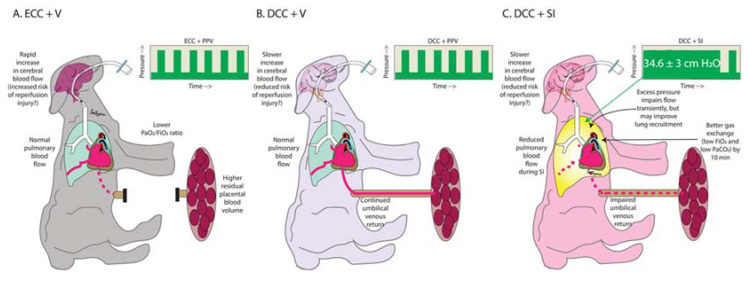
Summary: effects of (**A**) ECC + V—current practice, (**B**) DCC + V and (**C**) DCC + SI in our model of ovine asphyxia induced by cord occlusion.

In animal models and infants without asphyxia or with mild/moderate compromise, the benefits of physiological cord clamping (DCC after ventilation onset) are well established [9,14]. In our study, we compared DCC using two modes of ventilation, SI and PPV, which we compared to DCC without ventilation and ECC with immediate ventilation (current standard) in severely asphyxiated lambs. Few studies have evaluated DCC in a non-ventilated asphyxiated animal model. Delayed cord clamping for 60 s with ventilation mitigated post resuscitation cerebral hyperemia, but did not offer other substantial hemodynamic advantages. SI transiently decreased pulmonary perfusion, but led to a better gas exchange by 10 min when used in conjunction with DCC (Figure 5).

SI as an initial ventilation strategy for lung recruitment has shown some benefits. A two-fold increase in inflation volume and FRC was described with SI in nine term asphyxiated newborns [15]. SI reduced the need for mechanical ventilation at 72 h [16] in preterm infants. Recently, the well-designed multicenter, randomized, SAIL trial evaluating SI in extremely preterm infants at birth was terminated early with infants in the SI group demonstrating increased early mortality [17], without any clear etiology. Interestingly, 15–20% of infants in both arms underwent 30 s DCC prior to SI, though their outcomes were not reported separately. A unique feature of our study is the evaluation of SI in combination with intact cord resuscitation. Contrary to our hypothesis, we could not demonstrate improved systemic or pulmonary hemodynamic outcomes with SI and DCC in an asphyxiated model. In fact, we report deleterious effects of SI on reducing PBF and UV flow, possibly due to an effect on intrathoracic pressure and/or diaphragmatic excursion [18] with an intact cord during the first few minutes after birth. However, these deleterious effects that we observed with a high pressure of 34.6 ± 3 cm H_2_O with SI may not be seen with lower pressures in a non-asphyxiated model, with or without an intact cord.

Several different time periods and inflation pressures were evaluated in SI. We chose a 30 s SI breath with a peak inflating pressure (PIP) of ~35 cm H_2_O and PEEP of 5 cm H_2_O on the basis of prior studies in a similar model. Klingenberg et al. compared five 3 s inflations or a single 30 s inflation in resuscitation of near-term asphyxiated lambs with subsequent ventilation using inflations at 0.5 s at 60/min for all groups [6]. They noted earlier cardiovascular recovery (time taken to achieve a HR > 120/min) by almost 60 s in the single 30 s sustained inflation group compared to the no SI groups. We could not demonstrate a similar benefit to SI with either early or delayed cord clamping in our study; however, the incidence of cardiac arrest in our model complicated the analysis of this data.

While no differences in gas exchange were noted at 2 and 5 min, we demonstrated differences at 10 min (Table 3). We suspect that SI resulted in better recruitment of the lung establishing FRC, resulting in higher PaO_2_ values. In addition, a combination of SI and DCC enabled “dual-ventilation” during the first 5 min, leading to normocapnia. Hypercapnia is known to enhance V/Q mismatch [19]. We speculate that lambs in the DCC + SI arm achieved a higher P/F ratio due to better alveolar recruitment and V/Q matching secondary to normocapnia (Table 3).

The interplay between different modes of ventilation and timing of cord clamping has not been evaluated before and is a strength of our study. There is insufficient evidence for or against DCC in infants requiring resuscitation [11]. Currently, most information on intact cord resuscitation in asphyxia comes from translational ovine models similar to our current study. DCC for 15 min restored cardiac output and oxygenation and mitigated post-asphyxial rebound hypertension after resuscitation in a non-arrest ovine model of perinatal asphyxia induced by maternal iliac artery ligation [13]. In lambs with asphyxial arrest, DCC for 10 min caused significant reductions in post-asphyxial rebound hypertension, cerebral blood flow and oxygenation [20]. These studies indicate improved hemodynamics with a longer DCC duration, and along with a difference in methodology of induced asphyxia (cord occlusion vs. maternal iliac artery ligation), may explain lack of sustained benefits in our study. With a clinically feasible and widely adapted shorter duration of a DCC of 60 s, we demonstrated the mitigation of post-asphyxial hyperemia in our model.

DCC is a reasonable option for term and preterm deliveries that do not require resuscitation. The negative intrathoracic pressure generated by a vigorous, crying neonate potentially assists placental transfusion [21], estimated at 20–40 mL/kg [22], and increases oxygen delivery by increasing arterial oxygen content and cardiac output [23]. However, PPV does not appear to enhance placental transfusion [24]. It is also unclear if placental transfusion occurs in cord occlusion-induced perinatal asphyxia, especially in infants are born via c-section. In our study, including all available lambs, we were unable to demonstrate any advantage to neonatal blood volume in lambs with delayed cord clamping, with and without ventilation. However, asphyxial arrest causes cessation of cord blood transfer. The non-arrested subgroup analysis showed reduced residual placental blood volume with delayed cord clamping, suggesting that placental transfusion does occur in this cohort.

We acknowledge several limitations of our study including obvious species differences. The use of maternal general anesthesia, fetal instrumentation and cesarean section could influence the results. However, these variables were common to all groups and between-group differences in results can be at least partly attributable to the randomized intervention. Unanticipated arrest was the biggest drawback of our model. As arrest occurred subsequent to the intervention period, we included them as an intent-to-treat analysis. The interventions may have contributed to progressive asphyxia and asystole. CC and epinephrine could affect post resuscitation hemodynamics, leading to large variation in results. Analyzing primary outcomes in the non-arrest subgroup was difficult due to small numbers, and these subgroup results are reported in the Supplementary Material (Table S1), where the current practice (ECC + V) resulted in a rapid return to baseline CBF. However, our unique translational model addresses important unanswered questions, with this study demonstrating complex interactions between ventilation techniques and cord clamp timing.

## 5. Conclusions

We conclude that higher airway pressures delivered by a 30 s SI breath in near-term asphyxiated lambs with DCC reduced both UV and PBF without affecting UA or CBF. A significant reduction in PBF with SI persists in the first few minutes after birth. We speculate that reduced PBF with SI may lead to delayed pulmonary vascular transition in this asphyxiated newborn model, already at increased risk for pulmonary hypertension/respiratory failure. However, DCC + SI resulted in lower CO_2_ and a higher PaO_2_/FiO_2_ ratio. On the basis of our findings, we suggest that SI as a ventilation strategy, especially in combination with DCC, should be used with caution in asphyxia. Our findings reinforce the need for randomized clinical trials to evaluate optimal cord management and ventilation strategies in asphyxiated newborns.

## Figures and Tables

**Table 1:** Baseline Characteristics.

	ECC + V (*n* = 5)	ECC + SI (*n* = 6)	DCC (*n* = 5)	DCC + V (*n* = 5)	DCC + SI (*n* = 7)
Weight (kg)	4.1 ± 0.8	4.3 ± 1	3.7 ± 0.8	3.6 ± 0.4	3.7 ± 0.4
Male	2	2	3	3	5
Multiple gestation	3	3	3	4	4
Incidence of arrest (epinephrine doses)	2 (1)	3 (2)	2 (2)	2 (2)	3 (1)
Baseline hemodynamics					
Left carotid blood flow (mL/kg/min)	28 ± 14	33 ± 10	24 ± 11	26 ± 11	33 ± 9
Mean systemic blood pressure (mmHg)	49	45	43	41	49
Mean umbilical venous blood flow (mL/kg/min)	29.8 ± 20	28.4 ± 17	34.2 ± 14.3	34.7 ± 11.1	49.2 ± 12.7
Mean umbilical arterial blood flow (mL/kg/min)	34.3 ± 29.6	22 ± 9	32.6 ± 9.8	31.4 ± 11.4	40.1 ± 18.5
Asphyxia blood gas					
pH	6.86 ± 0.1	6.88 ± 0.1	6.89 ± 0.0	6.88 ± 0.1	6.88 ± 0.1
pCO_2_ (mm Hg)	111 ± 19	121 ± 11	123 ± 4	112 ± 34	119 ± 25
Lactate (mmol/L)	8.5 ± 2.5	9.0 ± 2.2	8.9 ± 2.3	9.8 ± 2.1	12.4 ± 2

**Table 2:** Primary outcomes and hemodynamic parameters during the intervention period.

	ECC + V (*n* = 5)	ECC + SI (*n* = 6)	DCC (*n* = 5)	DCC + V (*n* = 5)	DCC + SI (*n* = 7)
**Primary Outcomes**					
**Time to reach baseline CBF (s)**	115 ± 124	190 ± 83	295 ± 180	246 ± 246	207 ± 119
**Time to HR > 100 bpm (s)**	149 ± 101	151 ± 116	247 ± 161	243 ± 212	173 ± 126
**Time to mean BP > 40 mmHg (s)**	204 ± 189	217 ± 78	355 ± 181	281 ± 224	192 ± 107
**During intervention**					
**Airway pressure (cmH_2_O)**	19.5 ± 5	37.7 ± 3	n/a	21.8 ± 6	34.6 ± 3 **
**CBF (mean ± SD) mL/kg/min**	12.1 ± 11.6	8.1 ± 10.8	4.9 ± 3.7	4.9 ± 2.7	6.7 ± 8.2
**PBF (mean ± SD) mL/kg/min**	2.1 ± 4.1	5.0 ± 6.7	3.6 ± 4.4	6.6 ± 6.4	1.3 ± 3.1 *
**UV flow (mean ± SD) mL/kg/min**	n/a	n/a	4.5 ± 3.4	2.9 ± 2.2	1.6 ± 3.6 *
**UA flow (mean ± SD) mL/kg/min**	n/a	n/a	3.1 ± 2.5	0.7 ± 1.6	1.3 ± 3.4
**Mean BP (mean ± SD) mmHg**	25 ± 13	21 ± 4	25 ± 6	21 ± 7	20 ± 9

Values are presented as mean ± SD; * *p* < 0.05 by Kruskal–Wallis one-way analysis of variance between DCC groups; ** *p* < 0.005 by Kruskal–Wallis one-way analysis of variance between groups.

**Table 3:** Gas exchange parameters.

	ECC + V (*n* = 5)	ECC + SI (*n* = 6)	DCC (*n* = 5)	DCC + V (*n* = 5)	DCC + SI (*n* = 6)
Blood gas parameters at 2 min					
pH	6.9 ± 0.1	6.9 ± 0.1	6.9 ± 0.1	7.0 ± 0.2	6.9 ± 0.1
pCO_2_ (mmHg)	107 ± 16	110 ± 14	130 ± 24	87 ± 24	92 ± 26
pO_2_ (mmHg)	29 ± 24	27 ± 6	22 ± 14	29 ± 11	20 ± 8
FiO_2_	0.5 ± 0.4	0.8 ± 0.3	0.5 ± 0.3	0.5 ± 0.4	0.8 ± 0.4
P/F ratio	106 ± 133	41 ± 24	51 ± 38	79 ± 65	39 ± 38
Lactate (mmol/L)	8.3 ± 1.8	8.7 ± 1.5	10.2 ± 2.4	8.9 ± 1.5	11.7 ± 2.5
Blood gas parameters at 5 min					
pH	6.9 ± 0.2	6.9 ± 0.1	6.9 ± 0.1	6.9 ± 0.2	7 ± 0.2
pCO_2_ (mmHg)	102 ± 27	95 ± 11	99 ± 39	90 ± 42	66 ± 36
pO_2_ (mmHg)	61 ± 51	85 ± 50	37 ± 31	46 ± 30	45 ± 17
FiO_2_	0.6 ± 0.4	0.7 ± 0.3	0.6 ± 0.3	0.4 ± 0.2	0.5 ± 0.4
P/F ratio	126 ± 132	138 ± 60	93 ± 106	149 ± 110	127 ± 113
Lactate (mmol/L)	8.9 ± 2.3	8.8 ± 1.9	9.4 ± 3.4	9.8 ± 3.8	11.2 ± 3
Blood gas parameters at 10 min					
pH	7.0 ± 0.2	6.9 ± 0.1	6.9 ± 0.1	6.9 ± 0.2	7.0 ± 0.1
pCO_2_ (mmHg)	81 ± 15	82 ± 27	83 ± 39	73 ± 34	44 ± 15 *
pO_2_ (mmHg)	82 ± 36	106 ± 68	52 ± 30	76 ± 49	134 ± 80
FiO_2_	0.5 ± 0.3	0.5 ± 0.3	0.8 ± 0.4	0.7 ± 0.4	0.3 ± 0.1
P/F ratio	180 ± 72	227 ± 165	98 ± 83	165 ± 156	400 ± 151 *#
Lactate (mmol/L)	8.5 ± 2.6	8 ± 2.1	10 ± 3.7	9.1 ± 2.5	11.8 ± 3.3

* *p* < 0.05 by Kruskal–Wallis test of variance in all groups; # *p* < 0.02 Kruskal–Wallis test of variance in DCC groups.

**Table 4:** Measurement of blood volume and placental transfusion.

**A. All Lambs**	**ECC (*n* = 9)**	**DCC (*n* = 10)**
Fetoplacental blood volume (mL/kg)	82 ± 20	76 ± 18
Newborn blood volume (mL/kg)	61 ± 14	60 ± 17
Residual placental blood volume (mL/kg)	21 ± 15	16 ± 13
Fraction of fetoplacental volume in newborn	0.76 ± 0.1	0.8 ± 0.2
**B. Non-arrested**	**ECC (*n* = 5)**	**DCC (*n* = 7)**
Fetoplacental blood volume (mL/kg)	92 ± 18	74 ± 19
Newborn blood volume (mL/kg)	58 ± 13	58 ± 12
Residual placental blood volume (mL/kg)	35 ± 8	16 ± 14 *
Fraction of fetoplacental volume in newborn	0.65 ± 0.1	0.84 ± 0.2 *

Values are presented as mean ± SD * *p* < 0.05 by Mann–Whitney U test vs. ECC.

## Data Availability

The data presented in this study are available in this article.

## Supplementary Materials

The following are available online at https://www.mdpi.com/article/10.3390/children8050353/s1, Table S1: Subgroup without Arrest—Primary Outcomes.

## References

[1] Nair J., Kumar V.H.S. (2018). Current and emerging therapies in the management of hypoxic ischemic encephalopathy in neonates. Children.

[2] Saugstad O.D. (2011). Reducing global neonatal mortality is possible. Neonatology.

[3] Vento M., Saugstad O.D. (2010). Resuscitation of the term and preterm infant. Semin. Fet. Neonatal Med..

[4] Ersdal H.L., Mduma E., Svensen E., Perlman J.M. (2012). Early initiation of basic resuscitation interventions including face mask ventilation may reduce birth asphyxia related mortality in low-income countries: A prospective descriptive observational study. Resuscitation.

[5] Madar J., Roehr C.C., Ainsworth S., Ersdal H., Morley C., Rudiger M., Skare C., Szczapa T., Te Pas A., Trevisanuto D. (2021). European resuscitation council guidelines 2021: Newborn resuscitation and support of transition of infants at birth. Resuscitation.

[6] Klingenberg C., Sobotka K.S., Ong T., Allison B.J., Schmolzer G.M., Moss T.J., Polglase G.R., Dawson J.A., Davis P.G., Hooper S.B. (2013). Effect of sustained inflation duration; resuscitation of near-term asphyxiated lambs. Arch. Dis. Child. Fetal. Neonatal. Ed..

[7] Schmolzer G.M., O’Reilly M., Labossiere J., Lee T.F., Cowan S., Qin S., Bigam D.L., Cheung P.Y. (2013). Cardiopulmonary resuscitation with chest compressions during sustained inflations: A new technique of neonatal resuscitation that improves recovery and survival in a neonatal porcine model. Circulation.

[8] Vali P., Chandrasekharan P., Rawat M., Gugino S., Koenigsknecht C., Helman J., Mathew B., Berkelhamer S., Nair J., Lakshminrusimha S. (2017). Continuous chest compressions during sustained inflations in a perinatal asphyxial cardiac arrest lamb model. Pediatr. Crit. Care Med..

[9] Bhatt S., Alison B.J., Wallace E.M., Crossley K.J., Gill A.W., Kluckow M., te Pas A.B., Morley C.J., Polglase G.R., Hooper S.B. (2013). Delaying cord clamping until ventilation onset improves cardiovascular function at birth in preterm lambs. J. Physiol..

[10] Vali P., Gugino S., Koenigsknecht C., Helman J., Chandrasekharan P., Rawat M., Lakshminrusimha S., Nair J. (2018). The perinatal asphyxiated lamb model: A model for newborn resuscitation. J. Vis. Exp..

[11] Aziz K., Lee H.C., Escobedo M.B., Hoover A.V., Kamath-Rayne B.D., Kapadia V.S., Magid D.J., Niermeyer S., Schmolzer G.M., Szyld E. (2020). Part 5: Neonatal resuscitation: 2020 american heart association guidelines for cardiopulmonary resuscitation and emergency cardiovascular care. Circulation.

[12] Hudson I.R., Cavill I.A., Cooke A., Holland B.M., Hoy T.G., Trevett D., Turner T.L., Wardrop C.A. (1990). Biotin labeling of red cells in the measurement of red cell volume in preterm infants. Pediatr. Res..

[13] Polglase G.R., Blank D.A., Barton S.K., Miller S.L., Stojanovska V., Kluckow M., Gill A.W., LaRosa D., Te Pas A.B., Hooper S.B. (2018). Physiologically based cord clamping stabilises cardiac output and reduces cerebrovascular injury in asphyxiated near-term lambs. Arch. Dis. Child. Fetal. Neonatal. Ed..

[14] Andersson O., Rana N., Ewald U., Malqvist M., Stripple G., Basnet O., Subedi K., Kc A. (2019). Intact cord resuscitation versus early cord clamping in the treatment of depressed newborn infants during the first 10 min of birth (nepcord III)—A randomized clinical trial. Matern. Health Neonatol. Perinatol..

[15] Vyas H., Milner A.D., Hopkin I.E., Boon A.W. (1981). Physiologic responses to prolonged and slow-rise inflation in the resuscitation of the asphyxiated newborn infant. J. Pediatr..

[16] Lista G., Boni L., Scopesi F., Mosca F., Trevisanuto D., Messner H., Vento G., Magaldi R., Del Vecchio A., Agosti M. (2015). Sustained lung inflation at birth for preterm infants: A randomized clinical trial. Pediatrics.

[17] Kirpalani H., Ratcliffe S.J., Keszler M., Davis P.G., Foglia E.E., Te Pas A., Fernando M., Chaudhary A., Localio R., van Kaam A.H. (2019). Effect of sustained inflations vs intermittent positive pressure ventilation on bronchopulmonary dysplasia or death among extremely preterm infants: The sail randomized clinical trial. JAMA.

[18] Brouwer E., Te Pas A.B., Polglase G.R., McGillick E.V., Bohringer S., Crossley K.J., Rodgers K., Blank D., Yamaoka S., Gill A.W. (2020). Effect of spontaneous breathing on umbilical venous blood flow and placental transfusion during delayed cord clamping in preterm lambs. Arch. Dis. Child. Fetal. Neonatal. Ed..

[19] Feihl F., Eckert P., Brimioulle S., Jacobs O., Schaller M.D., Melot C., Naeije R. (2000). Permissive hypercapnia impairs pulmonary gas exchange in the acute respiratory distress syndrome. Am. J. Respir. Crit. Care Med..

[20] Polglase G.R., Schmolzer G.M., Roberts C.T., Blank D.A., Badurdeen S., Crossley K.J., Miller S.L., Stojanovska V., Galinsky R., Kluckow M. (2020). Cardiopulmonary resuscitation of asystolic newborn lambs prior to umbilical cord clamping; the timing of cord clamping matters!. Front. Physiol..

[21] Katheria A.C., Rich W.D., Bava S., Lakshminrusimha S. (2019). Placental transfusion for asphyxiated infants. Front Pediatr..

[22] Linderkamp O. (1982). Placental transfusion: Determinants and effects. Clin. Perinatol..

[23] Katheria A.C., Lakshminrusimha S., Rabe H., McAdams R., Mercer J.S. (2017). Placental transfusion: A review. J. Perinatol..

[24] Creasy R.K., Drost M., Green M.V., Morris J.A. (1972). Effect of ventilation on transfer of blood from placenta to neonate. Am. J. Physiol..

