# Supplementary material for: Sustained Inflation Reduces Pulmonary Blood Flow during Resuscitation with an Intact Cord
Source: Children (Basel). 2021 Apr 29;8(5):353. doi: 10.3390/children8050353 (PMC8145980; doi:10.3390/children8050353)
Supplement: Supplementary file 1 [file children-08-00353-s001.zip › children-1193862-supplementary.pdf]

Table S1. Subgroup Without Arrest- Primary Outcomes.

	ECC+V (n=3)	ECC+SI (n=3)	DCC (n=3)	DCC+V (n=3)	DCC+SI (n=3)
Time to reach baseline Ca flow (sec)	25 ± 18*	138 ± 44	236±166	135 ± 129	131 ± 124
Time to HR>100 (sec)	92 ± 86	46 ± 21	138±87	125 ± 113	107 ± 66
Time to mean BP>40 mmHg(sec)	178 ± 261	180 ± 103	355±224	221 ± 263	123 ± 80
